# A cost-effective and customizable automated irrigation system for precise high-throughput phenotyping in drought stress studies

**DOI:** 10.1371/journal.pone.0198546

**Published:** 2018-06-05

**Authors:** Diego Ortiz, Alexander G. Litvin, Maria G. Salas Fernandez

**Affiliations:** 1 Department of Agronomy, Iowa State University, Ames, Iowa, United States of America; 2 Department of Horticulture, Iowa State University, Ames, Iowa, United States of America; Institute of Genetics and Developmental Biology Chinese Academy of Sciences, CHINA

## Abstract

The development of high-yielding crops with drought tolerance is necessary to increase food, feed, fiber and fuel production. Methods that create similar environmental conditions for a large number of genotypes are essential to investigate plant responses to drought in gene discovery studies. Modern facilities that control water availability for each plant remain cost-prohibited to some sections of the research community. We present an alternative cost-effective automated irrigation system scalable for a high-throughput and controlled dry-down treatment of plants. This system was tested in sorghum using two experiments. First, four genotypes were subjected to ten days of dry-down to achieve three final Volumetric Water Content (VWC) levels: drought (0.10 and 0.20 m^3^ m^-3^) and control (0.30 m^3^ m^-3^). The final average VWC was 0.11, 0.22, and 0.31 m^3^ m^-3^, respectively, and significant differences in biomass accumulation were observed between control and drought treatments. Second, 42 diverse sorghum genotypes were subjected to a seven-day dry-down treatment for a final drought stress of 0.15 m^3^ m^-3^ VWC. The final average VWC was 0.17 m^3^ m^-3^, and plants presented significant differences in photosynthetic rate during the drought period. These results demonstrate that cost-effective automation systems can successfully control substrate water content for each plant, to accurately compare their phenotypic responses to drought, and be scaled up for high-throughput phenotyping studies.

## Introduction

In recent years, advances in genotyping through next generation sequencing techniques have facilitated the generation of genetic marker information at large scale and decreasing costs [1, 2]. Therefore, plant genotyping is no longer the largest constraint in genetic studies. On the contrary, high-throughput phenotyping (HTP), i.e. the measurement of quantitative/qualitative traits in a large number of plants, requires intense resource allocation and faces multiple technical challenges [3].

Drought is one of the most important abiotic stresses that reduces crop yields in dryland agriculture. The response of plants to limited water conditions can be studied at different scales, i.e. molecule, cell, tissue, plant, plots or replicable fields, which requires a suitable control of the stress duration and intensity [4, 5]. There is a need to generate reproducible and homogeneous conditions in soil water content or evaporative demand to successfully discover genes/genomic regions related to stress response [6].

Numerous genetic, genomic and proteomic studies characterizing plant responses to drought failed to implement an appropriate phenotyping method to ensure a fair comparison among genotypes [7]. Studies investigating changes in gene expression or protein concentration are frequently performed after irrigation is withdrawn from plants [8, 9]. One of the major problems of this approach relies on the variable growth and water consumption rates frequently observed among a diverse set of genotypes, and thus, at any given time during the drought treatment, soil water content can be different between the lines/cultivars under investigation. This can lead to confounded effects between levels of stress and genotypes. To overcome this problem, several high-throughput phenotyping facilities have been developed with state-of-the-art technologies that can control stress conditions at the individual plant level, e.g. PHENOPSIS [10], and the Plant Accelerator [3]. Yet, the high cost of these facilities is a constraint that prevents their broad adoption by the scientific community. Sensor control systems offer methods for precision control of climatic and cultural practices such as irrigation in research, but can be cost prohibitive [11, 12]. The development of reliable and cost-effective irrigation systems that could be scaled up to achieve the high-throughput needed for large genetic-genomic studies would benefit many plant research communities and advance plant science especially in the disciplines of breeding and physiology.

Recently, the cost of sensors has declined partly due to the development of new technological alternatives of high precision analogs such as capacitance sensors [13, 11]. These technological advances have made accurate monitoring methods more affordable for the scientific community. Capacitance and resistive sensors are low cost and based on similar concepts, since they both measure the electrical conductivity across two electrodes [14, 11]. Even though resistive sensors provide the highest cost savings at approximately $5/unit compared to $90/unit for a capacitance sensor (EC-5; Decagon Devices, Pullman, WA), they are considered more qualitative than quantitative because their moisture measurements are less reliable [14]. Resistive sensors excite the probe with a precise voltage and measure the direct drop of conductivity across electrodes. While this provides a simple measure of substrate electrical conductivity, changes in salinity might introduce errors, reducing accuracy and repeatability. Capacitance or dielectric sensors can produce an oscillating excitation of the probe electrodes, shifting charges through the substrate and, when sensors and cables are well insulated, they provide a more consistent measurement within range of substrate salinity, temperature, and moisture [14, 15, 16]. Sensor output can be influenced by media type, density, and general salinity, requiring sensor calibration for the used media type and range of operating substrate moisture. Although manufacturers may provide general calibration curve equations to calculate substrate moisture from voltage measurements, a substrate specific calibration is recommended prior to use for increased accuracy [17, 18]. Suitable calibrations across a wide variety of media have been reported for capacitance sensors used in field soils [19], containerized soilless substrates [20], and poultry manure [21].

Other high-resolution substrate sensor technologies include the use of time-domain reflectometry (TDR), which is more applicable to a variety of uses, both in field and containerized plant production. These sensors work by creating pulsed electromagnetic outputs and a time response for a return signal [11, 22, 23]. While data are less variable from a TDR than a capacitance sensor, they are generally cost-prohibitive and require operator knowledge on their use [11, 24].

Gravimetric sensors are another alternative of cost-effective sensors for water monitoring based on weight [25, 26]. At irrigation, a pot placed on a sensor would report a heavy load, and through the course of evapotranspiration, the pot weight would be reduced and trigger irrigation at a particular weight threshold of the gravimetric sensor [26]. Considering that these sensors are based on the overall mass of the load, the exact determination of substrate moisture is difficult. As a plant increases in size, the sensor may not be able to distinguish the added plant mass from the amount of water in the pot, without precise programming to adjust for the plant mass increase, leading to irregular or insufficient irrigation [26]. This technical difficulty to implement an irrigation threshold is particularly complex when a treatment is consistently applied to genetically diverse plants. Therefore, substrate moisture sensors are available in many affordable versions, but the final price of these sensors are intrinsically tied to its mechanism of measurement and overall precision [14, 11].

The introduction of low-cost controllers [27], and diverse systems for automation and monitoring [28, 29, 30] have facilitated the adoption of automation and data loggers by a broader group of researchers. Therefore, alternative high-resolution data loggers are available today for the precision control of automation and turnkey readiness of research projects. Additionally, the use of sensors with high built-in resolution and/or embedded chips [16, 31], and an increase in “do-it-yourself” (DIY) electronics [32, 27] have provided opportunities to utilize more affordable electronics to work in tandem or standalone replacement to traditional automation systems.

Data loggers and automation systems vary in cost depending on the resolution of recorded measurements and the completeness of accessories to automate. Research data loggers such as CR1000 (Campbell Scientific, Logan, UT) can cost approx. $1,600 per unit, can take measurements from eight to 16 sensors, record the data, and control a switch of 5 or 12 VDC as an action [33]. A multiplexer, such as AM16/32B (approx. cost $600, Campbell Scientific, Logan, UT) might be needed to connect up to a maximum of 48 sensors [33]. For the irrigation control of more than five solenoids, a relay driver (e.g. SDM16ACDC from Campbell Scientific, Logan, UT; approx. cost $900) can be used to control relays for up to 16 separate actions, and three of them would be required per multiplexer. Power supplies are also needed for the specific action of the relay drivers and the data logger.

For a cost-effective solution, DIY projects using open source controllers have been developed, such as those with Arduino. Considering the DIY nature of these components, each part is parsed so that the data logger is an Arduino microcontroller (~$15 per unit), an SD card adapter for the actual logging of data (~$10 per unit), and an SD card to insert into the system (~$10). The cost difference with a CR1000 data logger also represents a difference in precision of the system’s resolution to read voltage, with the CR1000 having a 13-bit resolution for analog voltage measurements (32-bit internal core), while an Arduino Mega has a 10-bit. This three-bit difference, constituting a four-fold increase in resolution, can add substantial variation in data read by an Arduino versus a CR1000, regardless of the sensor accuracy and represents the biggest disparity between the two systems. Considering that the critical difference between research grade systems and DIY open source electronics relate mostly to computation precision of the data logger, a hybridization of systems could lead to high-resolution data logging and control with a reduced financial cost per overall system.

Therefore, our objectives were to: i) leverage novel technologies in sensors and low-cost controllers to develop a modular irrigation system that could be scaled up for high-throughput plant phenotyping; ii) develop an automated irrigation system that allows a controlled dry-down and ensures a similar stress condition in all plants, maintaining a target Volumetric Water Content (VWC) for each plant independently; and iii) test the reliability of the system in a diverse set of sorghum plants with variable sizes and photosynthetic rates.

## Materials and methods

### Growth conditions

#### Evaluation of the controlled dry-down efficacy and final VWC

Four sorghum accessions were exposed to drought stress: PI533882, PI533996, PI656019, and PI656119. These accessions were selected based on their contrasting plant size and photosynthetic rate under both optimal and cold temperature conditions, as previously reported [34]. Plants were grown in seedling trays in a greenhouse with temperature conditions of 28C day / 24°C night and a photoperiod of 16 h of supplemental light. After two weeks, twelve plantlets per accession were transferred to 6 L pots with Metro Mix 900 soilless substrate (Sun Gro Horticulture, Agawam, MA). The same amount of substrate was placed in each pot (5.5 L) to ensure the same water holding capacity. Plants were subsequently moved to a growth chamber (model PGW36T, capacity 11.28 m^3^, Percival Scientific, Perry, IA) and adapted to high light conditions during the following two weeks, from 450 to 1000 *μ*mol photons m^-2^ s^-1^ photosynthetic photon flux density (PPFD). Every day, light conditions were sequentially increased from 5 am to 8 am and decreased from 5 pm to 8 pm to simulate sunrise and sunset, respectively. Plants were fertilized manually as needed with Peters Excel Cal-Mag Fertilizer (15-5-15; The Scotts Co., Marysville, OH).

Thirty one days after planting, plants were subjected to three water treatments based on a target final VWC: 0.10, 0.20 and 0.30 m^3^ m^-3^. Within each treatment, a VWC threshold was established per day such that the substrate water content would decrease at a drying rate of 0.01 and 0.02 m^3^ m^-3^ per day in all pots and all genotypes to reach the target VWC of 0.20 and 0.10 m^3^ m^-3^, respectively, over a ten-day period.

#### Scaling up the irrigation system using diverse sorghum genotypes

Forty-two accessions were grown in conditions similar to the previously described experiment with one plant per genotype placed into each of the two growth chambers (total of 42 plants per chamber). After adaptation to high light conditions, 33-day-old plants were subjected to the following water treatments: 1) three days at control VWC (0.30 m^3^ m^-3^); 2) seven days of controlled dry-down, and 3) three days at drought conditions (VWC = 0.15 m^3^ m^-3^). Based on preliminary results, 0.15 m^3^ m^-3^ VWC was an adequate final VWC to maximize the variation in photosynthetic response to drought and all plants reached this target VWC in a seven-day dry-down period, with an average drying rate of 0.012 m^3^ m^-3^ per day. Plants were irrigated manually during the control days (saturation) to ensure that VWC was above 0.30 m^3^ m^-3^ in all cases, and the irrigation system was used during the controlled dry-down and drought periods.

### Biomass and photosynthesis measurements

In the first experiment to evaluate the efficacy of the system at small scale, three plants per genotype were sampled before the beginning of the drought treatment to obtain initial dry matter weights (DM_i_). At the end of the experiment, three plants per genotype and water treatment were harvested to estimate final dry matter weight (DM_f_). In all cases, plants were cut at the base of the stem and dried in an oven at 75°C until the sample attained a constant weight. Plant growth (PG) was estimated as the total dry matter accumulated between the beginning and the end of the experiment (DM_f_ −DM_i_). Water use efficiency (WUE) was calculated as PG divided by irrigated water volume (IWV, see definition below).

In the second experiment to scale up the irrigation system, leaf net carbon assimilation rate (*A*) and stomatal conductance (*g*_*s*_) rates were measured using three LI-6400XT gas exchange analyzers (LI-COR, Lincoln, NE). Data were obtained in three consecutive days during both the control and drought periods, between 9 am and 2 pm. Conditions in the LI-6400XT leaf cuvette were set to 400 ppm CO_2_, a flow of 300 μmol s^-1^ CO_2_, 50–60% relative humidity, and 1000 μmol photons m^-2^ s^-1^ PPFD. After placing the Li-Cor cuvette on the leaf for a minimum of two minutes, stability of multiple parameters was monitored. Data were recorded when the coefficient of variation of A, flow, and humidity were below 1.2%.

### Irrigation system

**Fig 1:**
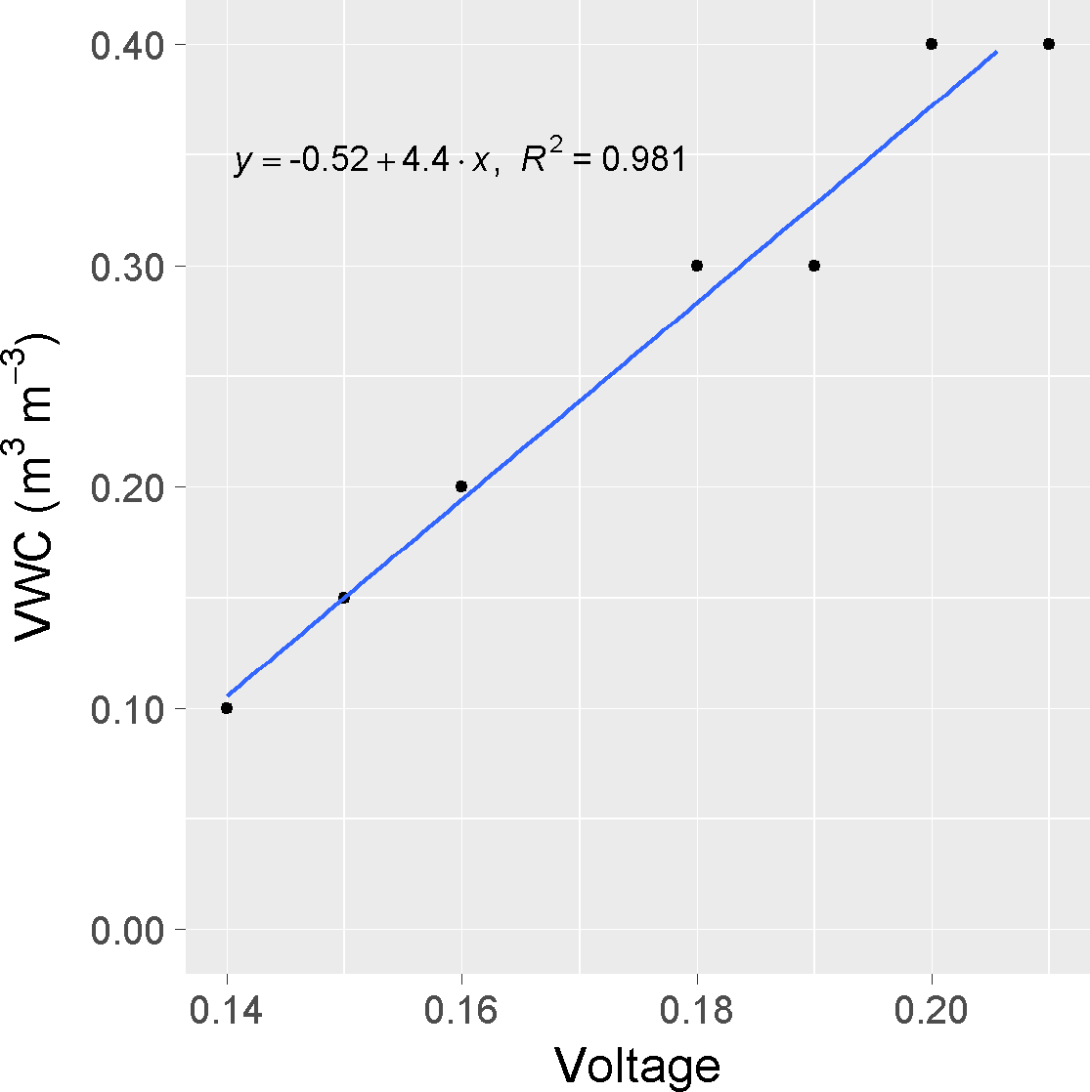
EC-5 sensor calibration for our specific soilless substrate. Volumetric water content (VWC) as a function of EC-5 sensor output voltage (mV). Individual replicates of voltage readings are plotted for each VWC. The sensor output voltage for the first three VWC were identical for both replications.

Substrate volumetric water content (VWC) was measured with capacitance sensors (EC-5; Decagon Devices, Pullman, WA), and calibrated specifically for this soilless substrate. Calibration entailed adding a measured amount of water to a fixed volume of oven-dried substrate in 500-ml containers to reach a target water content value. After homogenizing the soilless substrate, the sensor was placed, and voltage was recorded. Two replications were measured with independent sensors in each VWC level of 0.10, 0.15, 0.20, 0.30 and 0.40 m^3^ m^-3^. A simple linear regression equation was fit to VWC on voltage and the obtained parameters were used to estimate substrate water content in the irrigation system (Fig 1).

**Fig 2:**
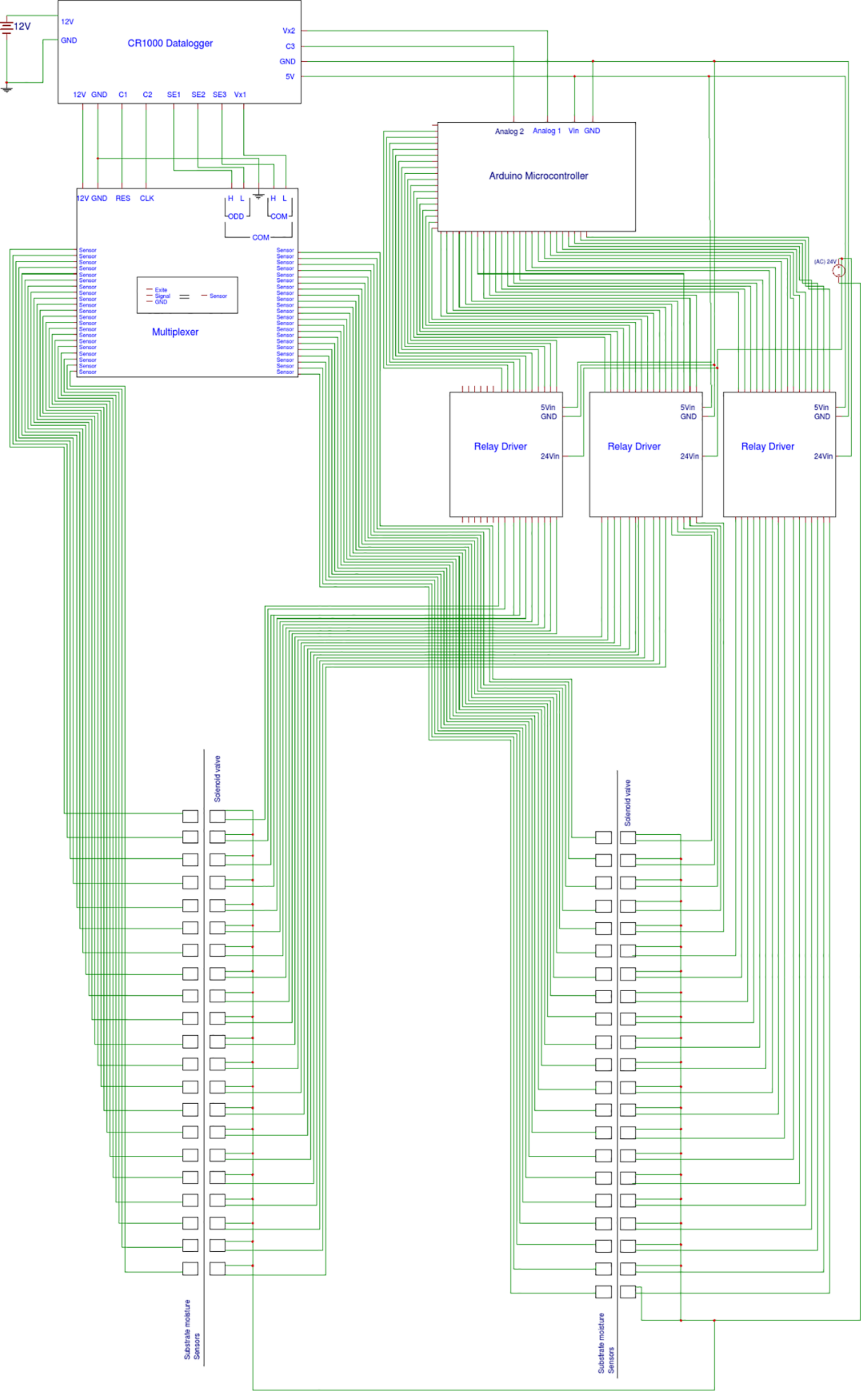
Automation system used for precision irrigation. Universal power supply signals designated by 24V, 12V, 5V, GND (ground), or Vin (Voltage in). Specific modules follow annotation for data logger ports (C ports: I/O control ports for 5VDC; SE ports: Single-ended communication ports for sensor signal data; Vx ports: Excitation variable voltage supply of 0–2.5V); Multiplexer (RES: Voltage supplied >3.5V sets active mode; CLK: Used to cycle through measurements of sensors; COM: Measurement terminals for ODD High (H), Low (L) and EVEN H, L for signal lead communication between sensors attached to multiplexer and data logger; Sensors: Individual excite, GND, and signal terminals for each sensor attached to the multiplexer); Microcontroller (Analog ports: Measures voltage signal from data logger of 0-5V to coordinate activation of irrigation signals to relay driver) Relay Drivers (Uses 5V signal inputs from microcontroller to activate 24V irrigation valves corresponding to respective sensor data).

**Fig 3:**
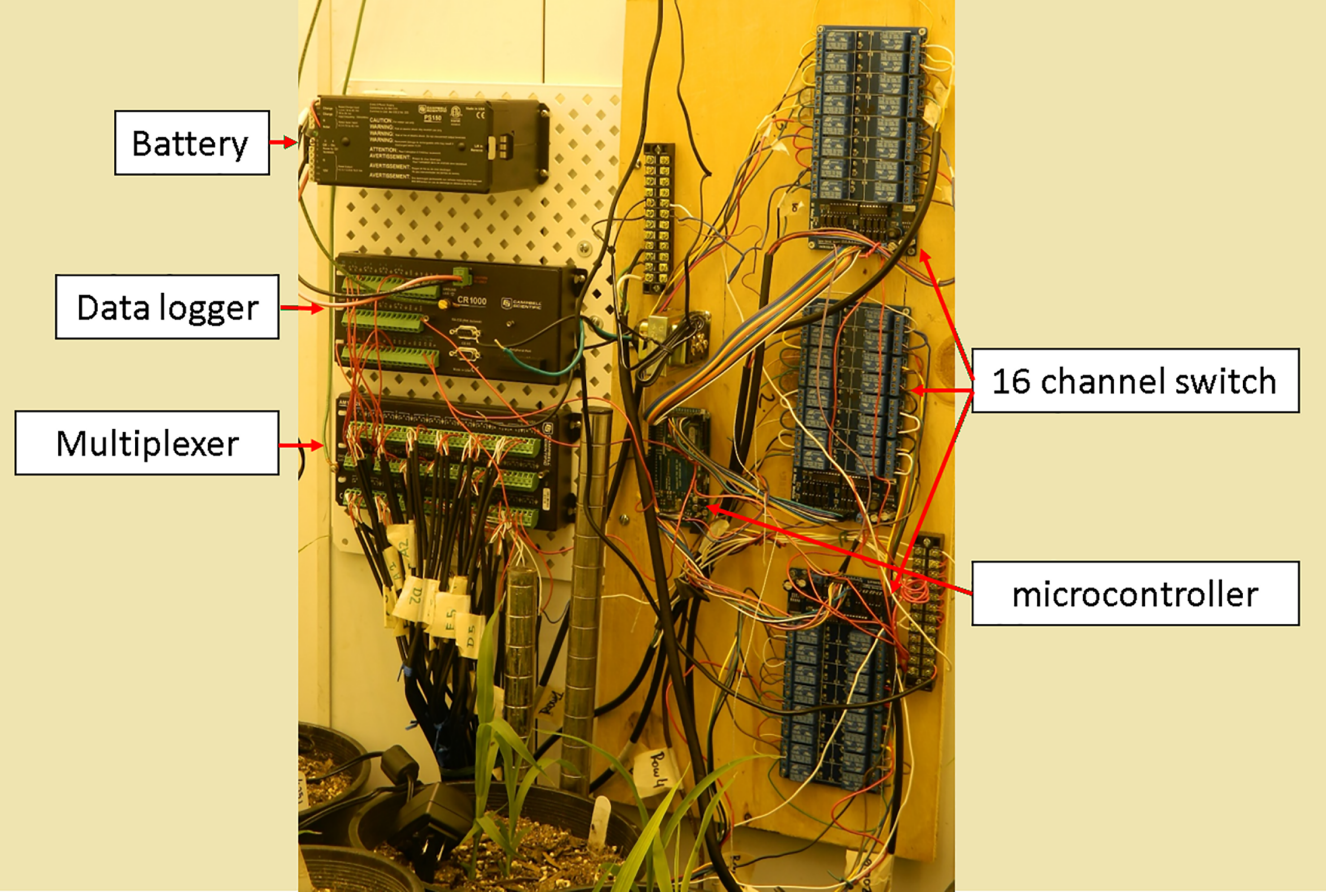
Irrigation system connections between data logger, multiplexer, microcontroller and 16 channel switch.

**Fig 4:**
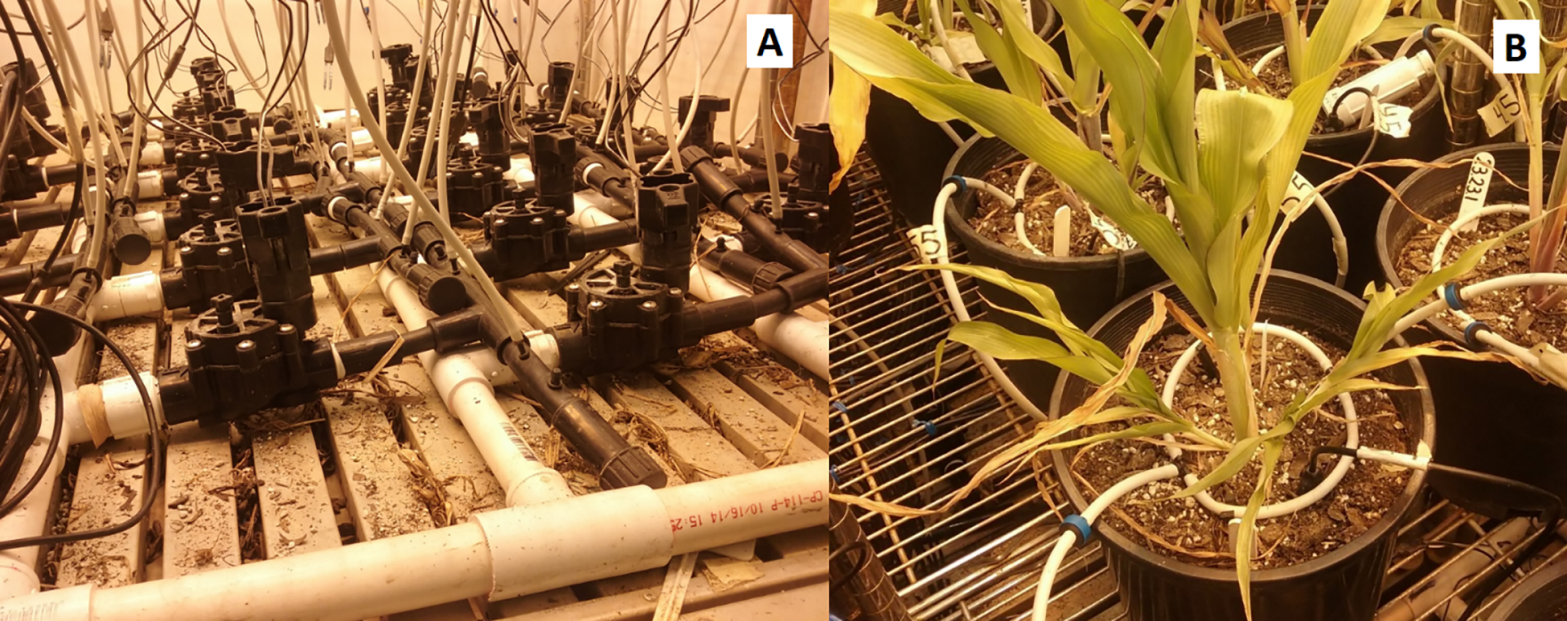
Arrangement and position of different components of the irrigation system. A) Solenoids and tubing; B) hose ring, EC-5 sensor and emitters.

One sensor was placed in each pot to individually control the VWC. Sensors were connected to a multiplexer (AM16/32B; Campbell Scientific, Logan, UT), and managed by a data logger (CR1000; Campbell Scientific) (Figs 2–4). The need for irrigation was determined by VWC set points programmed into the CR1000 and transmitted to a microcontroller (Mega 2560; Arduino, Ivrea, Italy) acting as a relay driver control for three 16 channel switches (SainSmart, Lenexa, KS). Once the CR1000 data logger measured and recorded VWC for each experimental unit, it generated a list of plants requiring irrigation. The data logger transmitted the list to the microcontroller operating the relay driver via a two channel pulse signaling with buffer arrays. One channel sent categorical identification of plant groupings, and the other sent voltage specific excitation signals corresponding to specific experimental units within each plant grouping. The concept for data transmission was based on a generalized principle of wave pipelining with self-reset logic to efficiently and quickly transmit data from the CR1000 following as reported by Litvin and Mourad [35]. The two channel one-way communication system from the Campbell CR1000 to the Arduino Mega used a pulsing I/O signal of either 0 or 5V, providing excitation of 5V to communicate onset of irrigation commands and cycle through grouped experimental units by cycling from 0V to 5V to initiate commands for the next group of experimental units. A second channel connecting the CR1000 to the Mega provided a voltage specific excitation from 0 to 2500 mV corresponding to a known experimental unit within that pulsed group. If a sensor recorded a VWC below the threshold, it would trigger the CR1000 to excite the channel to the respective voltage for irrigation need as reported by the sensor. The two channels worked in tandem, with the voltage variable channel only providing excitation during an active pulse (Fig 2).

The number of experimental units that could be reported per pulse is limited by the resolution of the data logger to provide a precise excitation voltage, and by the resolution of the microcontroller reading the voltage excitation. The accuracy and reliability of CR1000 to provide the excitation is high, but restricted by the Arduino’s 10-bit analog voltage resolution, which ultimately limited the amount of commands per 5V pulse. To mitigate this issue, groups were defined by pulses, which allowed commands corresponding to irrigation needs of experimental units to be spread across as many pulse signals as needed. Instead of setting a voltage equal to the expected excitation for a given experimental unit as a trigger for irrigation, the Arduino Mega associated ranges to encapsulate the expected value plus a tolerance in voltage in either direction. For example, if the CR1000 provided an excitation of 1800 mV, the Arduino Mega would recognize it as a signal of irrigation need since any voltage between 1600 and 2000 mV (1800 ± 200 mV) would trigger irrigation for the respective experimental unit. Spaces between acceptable voltage values for each experimental unit were undefined to prevent accidental triggering of a subsequent pot as a consequence of a voltage flux between commands. After the final pulse ended, the Arduino Mega ceased to “standby” for commands and began to cycle through irrigation events.

When irrigation was needed in a given pot, relay switches controlled by the Arduino Mega operated the solenoid valves (3/4” valve, RainBird, Azusa, CA) that corresponded to each sensor-controlled pot. Each pot was irrigated with two pressure-compensating emitters providing 1.89 L h^-1^ (Rain Bird, Azusa, CA). The program would measure VWC of each pot every 15 m (efficacy experiment) or 30 m (scaling up experiment) and if the respective threshold for an experimental unit’s VWC dropped below its set point, the corresponding irrigation valve was opened for a fixed amount of time per irrigation event. The duration of irrigation was adjusted individually for each solenoid to ensure that the water volume applied was 10–15 ml in the small-scale efficacy experiment and 20–30 ml when the system was scaled up to 42 pots per module. Data were recorded every 15 m (efficacy experiment) or 30 m (scaling-up experiment), at each run cycle, including the current VWC level and the number of executed irrigation events. Irrigated water volume (IWV) was calculated for each plant as the number of irrigation events multiplied by the applied water volume per solenoid. Once every 24 h, on the last run before midnight, data were summarized for each experimental unit as daily mean, minimum, and maximum VWC and the total amount of water used to irrigate each experimental unit.

### Statistical analysis

Data analyses were conducted using Proc GLM of SAS version 9.4 (SAS Institute, Cary, NC, USA). The first experiment (efficacy experiment) was a factorial design, with three water treatments, four accessions, and three replications (plants). The statistical model was:

$$Y_{ijk}=\mu +T_{i}+G_{i}+{TG}_{ij}+\varepsilon_{(ij)k}$$

where Y_ijk_ is the response variable, μ is the overall mean, T_i_ is the water treatment effect, G_j_ is the accession effect, TG_ij_ is the interaction between T and G, and ε_(ij)k_ is the residual. All treatments were considered fixed.

The second experiment (scaling-up experiment) was a randomized complete block design. Results for each water treatment period were analyzed separately, i.e. control and drought, and the statistical model was:

$$Y_{ij}=\mu +R_{i}+G_{j}+\varepsilon_{ij}$$

where Y_ij_ is the response variable (average of three days), μ is the overall mean, R_i_ is the replication (growth chamber) effect, G_j_ is the accession effect, and ε_ij_ is the residual. All treatments were considered random.

## Results

The calibration of sensors for the soilless substrate used in these experiments resulted in an accurate measurement of VWC (Fig 1). There was a linear relationship between the EC-5 sensor output and VWC, with a high R^2^ value of 0.98.

### Evaluation of the controlled dry-down efficacy and final VWC

**Fig 5:**
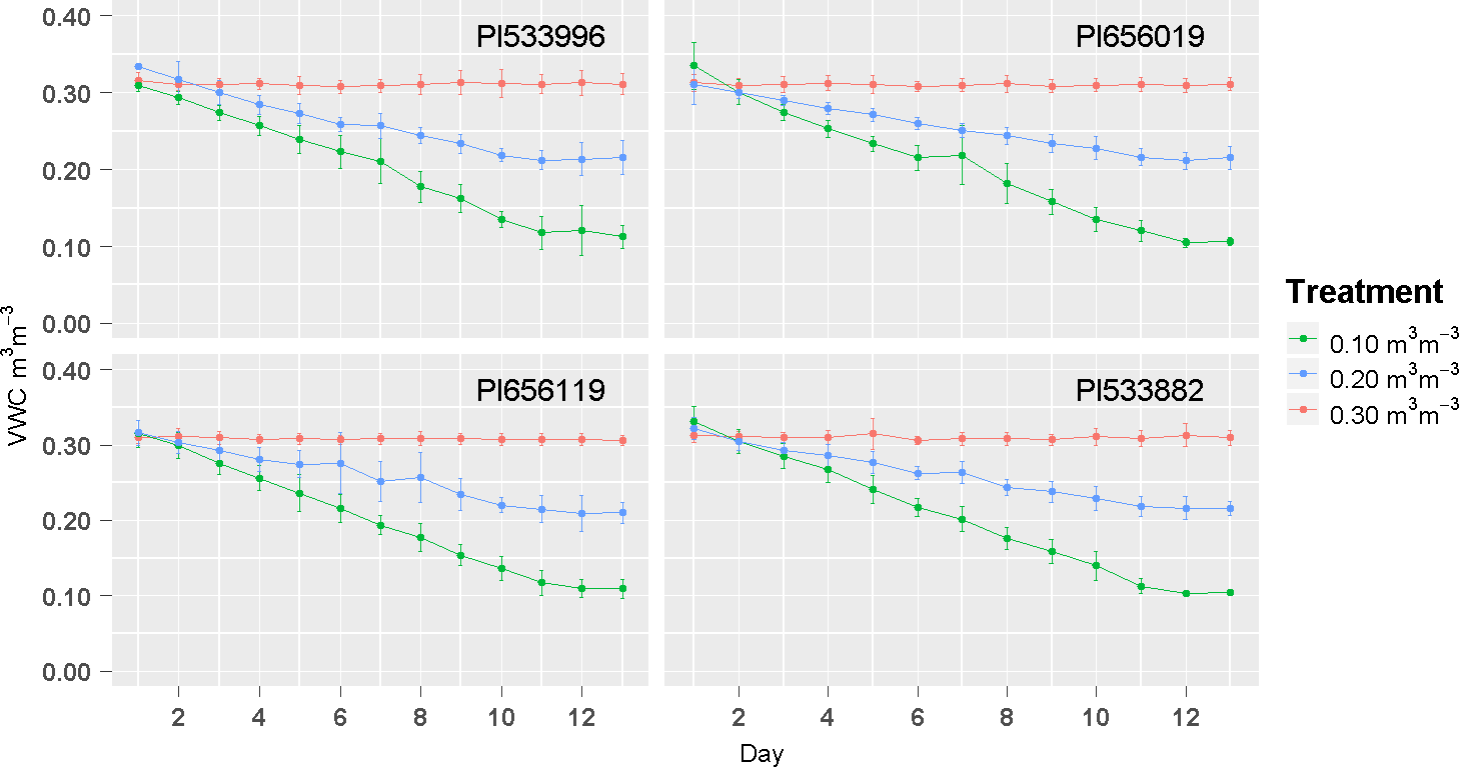
Changes in volumetric water content (VWC) over time using four sorghum genotypes subjected to three final VWC levels (0.10, 0.20 and 0.30 m^3^ m^-3^) after ten days of dry-down. Each point represents a daily VWC average. Bars indicate standard deviation.

The initial substrate VWC was similar in all plants, i.e. 0.31–0.36 m^3^ m^-3^, and decreased at expected rates in each treatment to reach the targeted VWC (Fig 5). Even though the four tested genotypes differed in final plant size (Table 1A and S1 Table), the irrigation system successfully controlled the substrate water content during the dry-down period and maintained it for three days during the drought treatment. The final average VWC was 0.11, 0.22, and 0.31 m^3^ m^-3^ with an average coefficient of variation of 8.7, 6.8, and 3.1%, respectively.

**Table 1:** Average dry matter per plant at the start (DM_i_) and last day (DM_f_) of water treatments, growth per plant (PG), irrigated water volume (IWV), and water use efficiency (WUE) in four sorghum genotypes.

A	Accession	DM_i_ (gr)	DM_f_ (gr)	PG (gr)	IWV (ml)	WUE
	PI656019	7.16 bc	19.21 b	16.07 a	1095.06 b	0.028 a
	PI656119	10.68 a	22.85 a	16.22 a	1766.72 a	0.017 b
	PI533882	6.01 c	12.31 d	8.4 b	631.28 b	0.024 ab
	PI533996	9.36 ab	15.57 c	8.28 b	828 b	0.024 ab
B	VWC Treatment (m^3^ m^-3^)	DM_i_ (gr)*	DM_f_ (gr)	PG (gr)	IWV (ml)	WUE
	0.10	8.3 a	17.6 b	9.29 b	992.33 a	0.024 a
	0.20	8.3 a	19.47 b	11.17 b	989.17 a	0.023 a
	0.30	8.3 a	24.57 a	16.27 a	1259.29 a	0.023 a

Plants were subjected to three final VWC levels (0.10, 0.20 and 0.30 m^3^ m^-3^) after ten days of controlled and constant dry-down. A) Average per genotype across VWC treatments (n = 9); B) Average across genotypes per VWC treatment (n = 12). Within columns, means with the same letter are not significantly different according to LSD (0.05).

* DMi for VWC treatments was calculated as the average across genotypes (n = 12) before the start of the dry-down period, and thus, the value is the same for all VWC treatments.

Accessions presented significant differences in initial and final plant biomass (*P*<0.05, Table 1A), as expected based on the selection criterion implemented to maximize variation in plant size and photosynthetic/transpiration capacity. The larger biomass accumulation (PG) observed for genotypes PI656019 and PI656119 (*P*<0.05) is explained by their larger canopy and plant size, including height and stem diameter (data not shown). Biomass accumulation was higher for plants grown under increasing VWC treatments, which demonstrates the ability of the irrigation system to control VWC. The total biomass accumulation (PG) obtained at the end of the 13 days of treatment was the highest under well-watered conditions (VWC = 0.30 m^3^ m^-3^). Even though plant growth was slightly lower for plants subjected to 0.10 m^3^ m^-3^ final VWC than those at 0.20 m^3^ m^-3^, there was no statistical difference between these treatments (Table 1B). When plants were exposed to 0.10 m^3^ m^-3^ final VWC, the total growth was reduced 43% compared to plants grown at 0.30 m^3^ m^-3^ VWC. Similarly, the 0.20 m^3^ m^-3^ VWC treatment resulted in plants that produced 31% less biomass than those under 0.30 m^3^ m^-3^ VWC. Both the irrigated water volume and WUE were significantly different among genotypes averaged over treatments, but not among water treatments over all genotypes (S1 Table, Table 1A and 1B). In general, irrigated water volume was highly and positively correlated with dry matter weight at the end of the treatment (DM_f_) and PG, but negatively correlated with WUE (Table 2). The effectiveness of the system to provide a specific water volume to each pot, according to the target VWC and the plant’s demand, is demonstrated by the IWV recorded for PI656119. This genotype had the largest plant size, a high growth rate (similar to PI656019) and required the largest water volume to be supplied over all treatments to maintain the target VWC.

**Table 2:** Correlation coefficients between all variables evaluated to determine the efficacy of the irrigation system to control the dry-down process and final VWC.

	DM_i_	DM_f_	PG	IWV	WUE
DM_i_	-				
DM_f_	0.51**	-			
PG	0.24	0.96***	-		
IWV	0.5**	0.76***	0.69***	-	
WUE	-0.29	-0.26	-0.2	-0.75***	-

Dry Matter (DM_i_), Final Dry matter (DM_f_), Plant growth (PG), Irrigated Water Volume (IWV) and Water Use Efficiency (WUE)

***Significant at *P* < 0.001

**Significant at *P* < 0.01

### Scaling up the irrigation system using diverse sorghum genotypes

To investigate the reliability of the system at a larger scale, we evaluated a set of 42 sorghum genotypes and compared their photosynthetic rate and stomatal conductance under both full irrigation (control) and drought conditions. We developed two independent modules of the irrigation system that were installed in two adjacent growth chambers used as replicates (Figs 2–4).

The system successfully controlled the substrate water content and all plants achieved the target VWC (0.15 m^3^ m^-3^) after seven days of dry-down, despite the significant differences among accessions observed in *g*_*s*_ when plants were subjected to drought stress (S2 Table, S3 Table, S1 Fig). The average VWC during the drought period ranged from 0.008 to 0.048 m^3^ m^-3^ above the target threshold (S4 Table).

## Discussion

The substrate specific calibration was fundamental to obtain accurate measurements in line with the manufacturer specifications for custom calibration [17, 18]. The linear relationship observed between the EC-5 output and VWC in the range of water content used herein is in agreement with previous studies, as demonstrated by the R^2^ values (0.92–0.98) [36, 37, 38].

When four genotypes were exposed to three target water levels, the observed variation in substrate water content was similar between treatments throughout the experiment (Fig 5), and was either within the sensor accuracy (±1–3%) [17, 18] or similar to the variation reported in other studies that investigated the performance of substrate moisture sensors for automated irrigation systems [39, 27]. The scaling up of the system using a larger and diverse set of genotypes resulted in a small range of variation in VWC during the drought period (0.008 to 0.048 m^3^ m^-3^ above the target threshold), in agreement with previous studies of automated systems based on capacitance sensors [28, 18]. This variation relative to the target VWC is lower than those of gravimetric methodologies [10] and thus, confirms the advantage of choosing this type of sensor during the design process of an irrigation system. In some cases, variation in VWC could be attributed to external factors, which include the inconsistent placement of emitters relative to sensors, and the movement of plants or sensors. Both of these factors could generate inaccurate readings and trigger additional unnecessary irrigations that would take VWC to levels above the target thresholds [27]. In this experiment, plants had to be occasionally moved to measure photosynthetic parameters with LI-6400XT and thus, the removal and repositioning of emitters and sensors could be causing the observed variation in VWC readings for each pot. However, these observed fluctuations were similar or less pronounced (1–5%) than those reported for other irrigation systems [27, 28], and more precise sensor and automation systems are often beyond the economic means for large-scale research [13, 11].

The design proposed herein facilitates the development of modular irrigation systems that could be deployed to independent growth chambers or utilized simultaneously in a large greenhouse setting. Therefore, the variability attributed to the independent function of each module (in this case each module corresponds to 42 pots) was an important parameter to evaluate. The observed average difference in VWC between replicates (or modules of the irrigation system) was 0.045 and 0.007 m^3^ m^-3^ for control and drought treatments, respectively (S4 Table), which demonstrates the reliability and accuracy of the independent modules. This result was confirmed by the comparative analysis of the physiological parameters obtained in this experiment. The mean difference in *A* and *g*_*s*_ between modules during the drought treatment was 2.21 *μ*mol CO_2_ m^-2^ s^-1^ and 0.005 mol H_2_0 m^-2^ s^-1^, respectively (S4 Table), which highlights the importance of generating similar stress conditions in each module for the correct characterization of the phenotype of interest [40].

Characterizing the drought stress response of diverse germplasm with drastic differences in photosynthetic capacity and stomatal conductance, such as the set used in the scaling up experiment (S2 Table, S4 Table), represents a major experimental challenge under controlled conditions. If the drought stress is simply imposed by withholding irrigation, the observed phenotypic responses would have confounded effects due to both true genotypic differences and those attributable to a variable stress level generated by uneven and non-comparable substrate water content among accessions. The stress conditions can be adjusted using our irrigation system to generate a fast or slow dry-down of plants over a longer or shorter period of time at a fixed VWC, depending on the objectives of the research. Both experiments demonstrate that this irrigation system can reach any target VWC within a broad range (0.10–0.30 m^3^ m^-3^ VWC) and can ensure a controlled dry-down period at a fixed rate for all plants over a variable time period (seven or ten days). Additionally, in some studies it is of special interest to impose the drought stress at a specific phenological stage, which results in some genotypes starting the dry-down at different days [10]. This adjustment can be done with the irrigation system presented herein, due to the control of VWC at the individual plant level.

There is a diverse array of methods to measure water content. In field experiments, the most common systems are based on neutron probes, gravimetric, and capacitance sensors [41, 4]. In growth chamber and greenhouse experiments, substrate water content is usually measured by weighing pots periodically, e.g. daily [42, 43], and by using dielectric sensors [28]. Although the gravimetric method is reliable, it requires frequent weighing and watering of plants to maintain a target VWC and is thus, very labor intensive. Furthermore, there is a need to sample extra plants regularly to quantify their biomass and then subtract those values from the total weight [10, 44]. Even though this method has been successfully used in small experiments [42, 43], more expensive platforms designed *ad hoc* are needed to implement it at large scale [10, 3, 45]. In general, these facilities are built *de novo*, which requires large funding resources, although some have been developed by adapting a pre-existing greenhouse [44, 46]. When systems based on capacitance sensors are used, there is no need to correct for plant weight as long as the sensors are properly calibrated [37]. One of the possible concerns of this type of irrigation system is the limited volume of influence of the dielectric sensors for accurate measurements. The EC-5 sensor selected for our irrigation system has been successfully used in experiments with small to medium pots [37], and in a wireless network of soil sensors deployed to the field [47, 38]. Considering that these sensors can overestimate VWC of large pots (i.e. 19 L) in dry-down conditions [48], sensors with a larger volume of influence could be incorporated in our design if large potted plants are needed, albeit increasing the overall cost of the system.

The need for characterizing plants at a large scale has driven the construction of several high-throughput phenotyping facilities [10, 3, 45]. Despite their great capabilities, the high costs associated with these large and stationary platforms make them inaccessible for many research groups. The irrigation system presented herein is an example of a modular, flexible and cost-effective design based on technological hybridizations. High precision processes were controlled by a data logger that can accurately excite and read substrate moisture sensors. The automation was determined by cost-saving microcontrollers and relay boards. New microcontrollers are constantly developed and, a sector once only populated by self-built circuits or Arduino, has opened now to an array of low-cost high-speed processing power that can be purchased with high-resolution analogs for precision instrumentation and data storage. Our system is also highly versatile, as it can be adapted to diverse greenhouse and growth chamber applications, or fieldwork with minor adjustments. Additionally, it could be improved or re-designed to include more complex capabilities by the incorporation of complementary sensor types, e.g. sensors of leaf temperature or light intensity, which makes it ideal for low-cost high-throughput phenotyping applications.

## Conclusions

We have developed and evaluated a modular irrigation system than can control VWC and stress levels at each individual pot and can be deployed for diverse applications and experimental conditions. The main advantages of our system include: i) the low cost of parts and sensors that makes it affordable for a larger scientific community compared to recently developed HTP platforms; ii) its adaptability to pre-existing growth chambers and greenhouse rooms; iii) the flexibility of the design that could incorporate additional sensors/cameras to suit specific phenotyping needs, and iv) its accuracy and reliability to impose a controlled dry-down period and specific final VWC even for mid-size pots. These features make this irrigation system a useful tool for phenotyping in plant breeding, genetic, genomic and physiological studies. Ultimately, all technological advances to accurately characterize plant responses to drought stress conditions will significantly contribute to the dissection of the genetic mechanisms controlling this important and complex trait.

## Supporting information

S1 Table Analysis of variance for Initial Dry Matter (DM_i_), Final Dry matter (DM_f_), Plant growth (PG), Irrigated Water Volume (IWV) and Water Use Efficiency (WUE), to evaluate the efficacy of the irrigation system to control the dry-down process and final VWC. (DOCX)

S2 Table Analysis of variance for photosynthetic rate (*A*) and stomatal conductance (*g*_*s*_) when the irrigation system is scaled up to phenotype diverse sorghum genotypes. (DOCX)

S3 Table Photosynthetic rate (*A*) and stomatal conductance (*g*_*s*_) in the control and drought treatments in 42 sorghum genotypes. *Values represent LS means and standard errors.
(DOCX)

S4 Table Average VWC in the control and drought treatments for 42 sorghum genotypes. Values in brackets represent standard deviation.
(DOCX)

S1 Fig Changes in volumetric water content over time after seven days of dry-down in the scaling up experiment. A subset of three genotypes and two replications is plotted as an example. Each point represents the average VWC throughout a day. The target final VWC was 0.15 m^3^ m^-3^. Day 0 = initial VWC before the start of the dry-down period.
(PNG)
