# Supplementary material for: A cost-effective and customizable automated irrigation system for precise high-throughput phenotyping in drought stress studies
Source: PLoS One. 2018 Jun 5;13(6):e0198546. doi: 10.1371/journal.pone.0198546 (PMC5988304; doi:10.1371/journal.pone.0198546)
Supplement: S1 Table — (DOCX) [file pone.0198546.s001.docx]

**S1 Table. Analysis of variance for Initial Dry Matter (DM_i_), Final Dry matter (DM_f_), Plant growth (PG), Irrigated Water Volume (IWV) and Water Use Efficiency (WUE)**, **to evaluate the efficacy of the irrigation system to control the dry-down process and final VWC.**

| **Source of variation** | **DM_i_** | |  | **DM_f_** | |  | **PG** | | |
| --- | --- | --- | --- | --- | --- | --- | --- | --- | --- |
|  | *F* Value | *p* value |  | *F* Value | *p* value |  | *F* Value | *p* value | |
| **T** |  |  |  | 20.58 | <.0001 |  | 20.58 | <.0001 | |
| **G** | 5.93 | 0.0197 |  | 36.99 | <.0001 |  | 24.09 | <.0001 | |
| **T*G** |  |  |  | 1.35 | 0.2735 |  | 1.35 | 0.2735 | |
| **Source of variation** | **IWV** | |  | **WUE** | |  |  |  |  |
|  | *F* Value | *p* value |  | *F* Value | *p* value |  |  |  |  |
| **T** | 1.02 | 0.376 |  | 0.57 | 0.5725 |  |  |  |  |
| **G** | 6.99 | 0.0015 |  | 2.42 | 0.0912 |  |  |  |  |
| **T*G** | 0.21 | 0.9714 |  | 0.3 | 0.9323 |  |  |  |  |

Treatment (T), Genotype (G) and the genotype by treatment interaction were treated as fixed effects.
