# Supplementary material for: A cost-effective and customizable automated irrigation system for precise high-throughput phenotyping in drought stress studies
Source: PLoS One. 2018 Jun 5;13(6):e0198546. doi: 10.1371/journal.pone.0198546 (PMC5988304; doi:10.1371/journal.pone.0198546)
Supplement: S2 Table — (DOCX) [file pone.0198546.s002.docx]

**S2 Table. Analysis of variance for photosynthetic rate (*A*) and stomatal conductance (*g_s_*) when the irrigation system is scaled up to phenotype diverse sorghum genotypes.**

| Source of variation |  | ***A* control** | |  | ***A* drought** | |  | ***g_s_* control** | |  | ***g_s_* drought** | |
| --- | --- | --- | --- | --- | --- | --- | --- | --- | --- | --- | --- | --- |
|  |  | *F* Value | *p* value |  | *F* Value | *p* value |  | *F* Value | *p* value |  | *F* Value | *p* value |
| Rep |  | 11.97 | 0.0013 |  | 8.34 | 0.0062 |  | 0.53 | 0.4706 |  | 2.02 | 0.1628 |
| Geno |  | 1.71 | 0.0443 |  | 1.81 | 0.0299 |  | 1.33 | 0.1823 |  | 1.7 | 0.0462 |

Rep: replication effect. Geno: genotype effect
