# Supplementary material for: A cost-effective and customizable automated irrigation system for precise high-throughput phenotyping in drought stress studies
Source: PLoS One. 2018 Jun 5;13(6):e0198546. doi: 10.1371/journal.pone.0198546 (PMC5988304; doi:10.1371/journal.pone.0198546)
Supplement: S3 Table — *Values represent LS means and standard errors. (DOCX) [file pone.0198546.s003.docx]

**S3 Table. Photosynthetic rate (*A*) and stomatal conductance (*g_s_*) in the control and drought treatments in 42 sorghum genotypes.** *Values represent LS means and standard errors.

|  |  | Control | |  | Drought | |
| --- | --- | --- | --- | --- | --- | --- |
|  |  | ***A**** | ***g_s_* *** |  | ***A**** | ***g_s_* *** |
| Genotype |  | *μ*mol CO_2_ m^-2^ s^-1^ | mol H_2_0 m^-2^ s^-1^ |  | *μ*mol CO_2_ m^-2^ s^-1^ | mol H_2_0 m^-2^ s^-1^ |
| PI533758 |  | 33.56 (1.45) | 0.184 (0.011) |  | 13.57 (2.42) | 0.058 (0.012) |
| PI533761 |  | 32.77 (1.45) | 0.178 (0.011) |  | 18.88 (2.42) | 0.087 (0.012) |
| PI533769 |  | 32.73 (1.45) | 0.161 (0.011) |  | 11.37 (2.42) | 0.044 (0.012) |
| PI533788 |  | 31.46 (1.45) | 0.166 (0.011) |  | 9.35 (2.42) | 0.039 (0.012) |
| PI533839 |  | 30.83 (1.45) | 0.157 (0.011) |  | 14.8 (2.42) | 0.063 (0.012) |
| PI533852 |  | 35.12 (1.45) | 0.189 (0.011) |  | 13.71 (2.42) | 0.059 (0.012) |
| PI533938 |  | 34.24 (1.45) | 0.188 (0.011) |  | 15.43 (2.42) | 0.067 (0.012) |
| PI533940 |  | 32.91 (1.45) | 0.172 (0.011) |  | 15.78 (2.42) | 0.071 (0.012) |
| PI533970 |  | 37.98 (1.45) | 0.202 (0.011) |  | 19.36 (2.42) | 0.083 (0.012) |
| PI533979 |  | 35.79 (1.45) | 0.199 (0.011) |  | 10.9 (2.42) | 0.049 (0.012) |
| PI533985 |  | 30.89 (1.45) | 0.164 (0.011) |  | 6.14 (2.42) | 0.026 (0.012) |
| PI534009 |  | 34.11 (1.45) | 0.182 (0.011) |  | 11.45 (2.42) | 0.05 (0.012) |
| PI534070 |  | 34.14 (1.45) | 0.188 (0.011) |  | 13.03 (2.42) | 0.057 (0.012) |
| PI534079 |  | 33.29 (1.45) | 0.178 (0.011) |  | 19.38 (2.42) | 0.085 (0.012) |
| PI534096 |  | 33.17 (1.45) | 0.166 (0.011) |  | 15.29 (2.42) | 0.064 (0.012) |
| PI534138 |  | 35.03 (1.45) | 0.182 (0.011) |  | 18.17 (2.42) | 0.08 (0.012) |
| PI561071 |  | 33.92 (1.45) | 0.19 (0.011) |  | 12.9 (2.42) | 0.055 (0.012) |
| PI564163 |  | 34.55 (1.45) | 0.173 (0.011) |  | 19.82 (2.42) | 0.082 (0.012) |
| PI576347 |  | 31.89 (1.45) | 0.159 (0.011) |  | 10.38 (2.42) | 0.042 (0.012) |
| PI576391 |  | 36.05 (1.45) | 0.193 (0.011) |  | 11.02 (2.42) | 0.046 (0.012) |
| PI576435 |  | 30.51 (1.45) | 0.168 (0.011) |  | 13.66 (2.42) | 0.059 (0.012) |
| PI597945 |  | 33.37 (1.45) | 0.168 (0.011) |  | 9.22 (2.42) | 0.037 (0.012) |
| PI597960 |  | 32.56 (1.45) | 0.162 (0.011) |  | 12.9 (2.42) | 0.052 (0.012) |
| PI597961 |  | 29.93 (1.45) | 0.161 (0.011) |  | 11.82 (2.42) | 0.05 (0.012) |
| PI597971 |  | 35.61 (1.45) | 0.182 (0.011) |  | 13.5 (2.42) | 0.061 (0.012) |
| PI598069 |  | 32.69 (1.45) | 0.181 (0.011) |  | 12.38 (2.42) | 0.052 (0.012) |
| PI601816 |  | 34.85 (1.45) | 0.185 (0.011) |  | 12.67 (2.42) | 0.048 (0.012) |
| PI655971 |  | 36.95 (1.45) | 0.191 (0.011) |  | 17.71 (2.42) | 0.072 (0.012) |
| PI655972 |  | 33.55 (1.45) | 0.176 (0.011) |  | 12.09 (2.42) | 0.051 (0.012) |
| PI655986 |  | 34.05 (1.45) | 0.188 (0.011) |  | 14.82 (2.42) | 0.064 (0.012) |
| PI655988 |  | 34.18 (1.45) | 0.177 (0.011) |  | 11.23 (2.42) | 0.048 (0.012) |
| PI655996 |  | 36.76 (1.45) | 0.192 (0.011) |  | 8.22 (2.42) | 0.032 (0.012) |
| PI656017 |  | 33.1 (1.45) | 0.176 (0.011) |  | 10.45 (2.42) | 0.042 (0.012) |
| PI656028 |  | 34.49 (1.45) | 0.184 (0.011) |  | 11.84 (2.42) | 0.049 (0.012) |
| PI656029 |  | 32.19 (1.45) | 0.167 (0.011) |  | 15.29 (2.42) | 0.068 (0.012) |
| PI656031 |  | 33.5 (1.45) | 0.189 (0.011) |  | 18.06 (2.42) | 0.084 (0.012) |
| PI656037 |  | 32.83 (1.45) | 0.176 (0.011) |  | 11.8 (2.42) | 0.051 (0.012) |
| PI656044 |  | 35.24 (1.45) | 0.181 (0.011) |  | 16.28 (2.42) | 0.069 (0.012) |
| PI656051 |  | 30.53 (1.45) | 0.161 (0.011) |  | 10.6 (2.42) | 0.044 (0.012) |
| PI656074 |  | 37.15 (1.45) | 0.202 (0.011) |  | 13.54 (2.42) | 0.054 (0.012) |
| PI656076 |  | 31.21 (1.45) | 0.154 (0.011) |  | 10.9 (2.42) | 0.045 (0.012) |
| PI656106 |  | 32.82 (1.45) | 0.17 (0.011) |  | 9.12 (2.42) | 0.039 (0.012) |
