# Supplementary material for: A cost-effective and customizable automated irrigation system for precise high-throughput phenotyping in drought stress studies
Source: PLoS One. 2018 Jun 5;13(6):e0198546. doi: 10.1371/journal.pone.0198546 (PMC5988304; doi:10.1371/journal.pone.0198546)
Supplement: S4 Table — Values in brackets represent standard deviation. (DOCX) [file pone.0198546.s004.docx]

**S4 Table. Average VWC in the control and drought treatments for 42 sorghum genotypes.** Values in brackets represent standard deviation

|  |  | VWC control (m^3^ m^-3^) | |  | VWC drought (m^3^ m^-3^) | |
| --- | --- | --- | --- | --- | --- | --- |
| Genotype |  | Rep 1 | Rep 2 |  | Rep 1 | Rep 2 |
| PI533758 |  | 0.362 (0.03) | 0.323 (0.047) |  | 0.176 (0) | 0.195 (0.012) |
| PI533761 |  | 0.298 (0.039) | 0.327 (0.022) |  | 0.166 (0.007) | 0.172 (0.002) |
| PI533769 |  | 0.314 (0.009) | 0.259 (0.024) |  | 0.167 (0.008) | 0.184 (0.004) |
| PI533788 |  | 0.328 (0.009) | 0.304 (0.043) |  | 0.171 (0.009) | 0.183 (0.014) |
| PI533839 |  | 0.347 (0.009) | 0.301 (0.012) |  | 0.166 (0.007) | 0.179 (0.017) |
| PI533852 |  | 0.317 (0.028) | 0.302 (0.022) |  | 0.178 (0.001) | 0.178 (0.014) |
| PI533938 |  | 0.328 (0.053) | 0.356 (0.061) |  | 0.182 (0) | 0.186 (0.012) |
| PI533940 |  | 0.396 (0.065) | 0.328 (0.009) |  | 0.178 (0.001) | 0.19 (0.012) |
| PI533970 |  | 0.396 (0.032) | 0.319 (0.044) |  | 0.171 (0.004) | 0.174 (0.021) |
| PI533979 |  | 0.314 (0.042) | 0.356 (0.05) |  | 0.182 (0.005) | 0.178 (0.01) |
| PI533985 |  | 0.362 (0.026) | 0.308 (0.025) |  | 0.165 (0.008) | 0.169 (0.012) |
| PI534009 |  | 0.327 (0.038) | 0.302 (0.015) |  | 0.185 (0.003) | 0.171 (0.009) |
| PI534070 |  | 0.341 (0.024) | 0.299 (0.033) |  | 0.179 (0.002) | 0.174 (0.015) |
| PI534079 |  | 0.311 (0.022) | 0.359 (0.01) |  | 0.171 (0.006) | 0.189 (0.005) |
| PI534096 |  | 0.34 (0.033) | 0.343 (0.047) |  | 0.18 (0.003) | 0.185 (0.006) |
| PI534138 |  | 0.387 (0.025) | 0.304 (0.037) |  | 0.173 (0.008) | 0.172 (0.009) |
| PI561071 |  | 0.357 (0.016) | 0.292 (0.015) |  | 0.18 (0.005) | 0.172 (0.01) |
| PI564163 |  | 0.332 (0.037) | 0.283 (0.048) |  | 0.173 (0.002) | 0.185 (0.014) |
| PI576347 |  | 0.362 (0.067) | 0.32 (0.05) |  | 0.161 (0.004) | 0.181 (0.014) |
| PI576391 |  | 0.332 (0.057) | 0.321 (0.046) |  | 0.166 (0.021) | 0.183 (0.018) |
| PI576435 |  | 0.272 (0.017) | 0.305 (0.058) |  | 0.177 (0.006) | 0.181 (0.006) |
| PI597945 |  | 0.31 (0.035) | 0.312 (0.042) |  | 0.172 (0.005) | 0.199 (0.012) |
| PI597960 |  | 0.409 (0.039) | 0.278 (0.005) |  | 0.183 (0.004) | 0.187 (0.015) |
| PI597961 |  | 0.32 (0.009) | 0.333 (0.009) |  | 0.182 (0.01) | 0.18 (0.015) |
| PI597971 |  | 0.392 (0.019) | 0.335 (0.035) |  | 0.159 (0.002) | 0.184 (0.013) |
| PI598069 |  | 0.33 (0.016) | 0.285 (0.044) |  | 0.175 (0.01) | 0.172 (0.018) |
| PI601816 |  | 0.311 (0.049) | 0.303 (0.011) |  | 0.172 (0.01) | 0.189 (0.014) |
| PI655971 |  | 0.354 (0.014) | 0.298 (0.031) |  | 0.175 (0.009) | 0.181 (0.01) |
| PI655972 |  | 0.382 (0.059) | 0.275 (0.044) |  | 0.185 (0.006) | 0.169 (0.016) |
| PI655986 |  | 0.366 (0.033) | 0.324 (0.034) |  | 0.182 (0.004) | 0.18 (0.001) |
| PI655988 |  | 0.319 (0.045) | 0.32 (0.015) |  | 0.169 (0.012) | 0.183 (0.01) |
| PI655996 |  | 0.377 (0.038) | 0.319 (0.031) |  | 0.176 (0.001) | 0.172 (0.004) |
| PI656017 |  | 0.382 (0.038) | 0.326 (0.035) |  | 0.175 (0.018) | 0.189 (0.019) |
| PI656028 |  | 0.381 (0.053) | 0.27 (0.007) |  | 0.167 (0.009) | 0.179 (0.011) |
| PI656029 |  | 0.318 (0.03) | 0.336 (0.02) |  | 0.174 (0.005) | 0.163 (0.014) |
| PI656031 |  | 0.344 (0.02) | 0.262 (0.013) |  | 0.177 (0.009) | 0.196 (0.012) |
| PI656037 |  | 0.341 (0.011) | 0.359 (0.056) |  | 0.159 (0.032) | 0.174 (0.017) |
| PI656044 |  | 0.369 (0.019) | 0.308 (0.022) |  | 0.178 (0.001) | 0.17 (0.011) |
| PI656051 |  | 0.355 (0.028) | 0.324 (0.038) |  | 0.167 (0.005) | 0.171 (0.008) |
| PI656074 |  | 0.343 (0.028) | 0.321 (0.044) |  | 0.171 (0.001) | 0.175 (0.006) |
| PI656076 |  | 0.345 (0.034) | 0.322 (0.029) |  | 0.179 (0.003) | 0.197 (0.006) |
| PI656106 |  | 0.385 (0.023) | 0.317 (0.054) |  | 0.168 (0.006) | 0.188 (0.007) |
