# Supplementary figures and images for: A cost-effective and customizable automated irrigation system for precise high-throughput phenotyping in drought stress studies
Source: PLoS One. 2018 Jun 5;13(6):e0198546. doi: 10.1371/journal.pone.0198546 (PMC5988304; doi:10.1371/journal.pone.0198546)

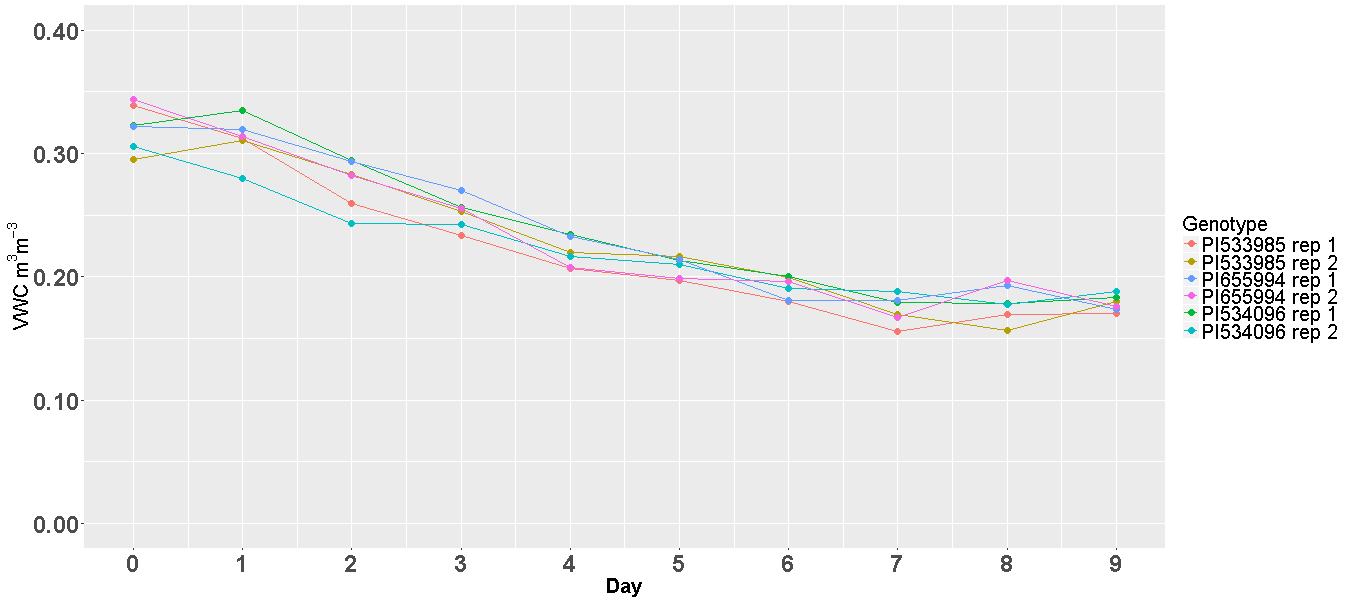

Supplement: S1 Fig — A subset of three genotypes and two replications is plotted as an example. Each point represents the average VWC throughout a day. The target final VWC was 0.15 m3 m-3. Day 0 = initial VWC before the start of the dry-down period. (PNG) [file pone.0198546.s005.png]
